# Single-Cell RNA-Sequencing Portraying Functional Diversity and Clinical Implications of IFI6 in Ovarian Cancer

**DOI:** 10.3389/fcell.2021.677697

**Published:** 2021-08-25

**Authors:** Hongyu Zhao, Zhefeng Li, Yan Gao, Jie Li, Xiaoting Zhao, Wentao Yue

**Affiliations:** Central Laboratory, Beijing Obstetrics and Gynecology Hospital, Capital Medical University, Beijing, China

**Keywords:** ovarian cancer, single-cell RNA-sequencing, heterogeneity, prognosis, IFI6, cisplatin resistance

## Abstract

Ovarian cancer (OC) is one of the most lethal gynecologic malignancies. Most patients die of metastasis due to a lack of other treatments aimed at improving the prognosis of OC patients. In the present study, we use multiple methods to identify prognostic S1 as the dominant subtype in OC, possessing the most ligand–receptor pairs with other cell types. Based on markers of S1, the consensus clustering algorithm is used to explore the clinical treatment subtype in OC. As a result, we identify two clusters associated with distinct survival and drug response. Notably, IFI6 contributes to the cluster classification and seems to be a vital gene in OC carcinogenesis. Functional enrichment analysis demonstrates that its functions involve G2M and cisplatin resistance, and downregulation of IFI6 suppresses proliferation capabilities and significantly potentiates cisplatin-induced apoptosis of OC cells *in vitro*. To explore possible mechanisms of IFI6 influencing OC proliferation and cisplatin resistance, GSEA is conducted and shows that IFI6 is positively correlated with the NF-κB pathway, which is validated by RT-qPCR. Significantly, we develop a prognostic model including IFI6, RiskScore, which is an independent prognostic factor and presents encouraging prognostic values. Our findings provide novel insights into elucidating the biology of OC based on single-cell RNA-sequencing. Moreover, this approach is potentially helpful for personalized anti-cancer strategies and predicting outcomes in the setting of OC.

## Introduction

Ovarian cancer (OC) is one of the most aggressive gynecological cancers among women worldwide, with an increased incidence in recent years. Despite the fact that much progress has been made toward OC treatment regarding surgery, chemotherapy, targeted therapy, and neoadjuvant chemotherapy, nearly 25% of OC patients are found to relapse within 6 months after combination therapy (Miller et al., 2009). Most patients die of metastasis due to a lack of other treatments aimed at improving the prognosis of OC patients. Thus, it is required to recognize OC-related risks and formulate optimal and effective therapeutic strategies.

Dysregulated signal transduction or genetic variation of tumor cells is confirmed to accelerate carcinogenesis (Karlberg et al., 2004; Bast et al., 2009). Efforts have been made to discuss the genomic changes in OCs and identify abnormal molecules that influence the pathophysiology, prognosis (Ramus et al., 2015; Millstein et al., 2020), and therapeutic targets (Sonego et al., 2019; Gralewska et al., 2020; Oza et al., 2020). However, the identified therapeutic targets based on bulk profiling technologies are not suitable for all patients, because of a complete disregard for intra-tumoral heterogeneity. Notably, intra-tumorigenic heterogeneity is a key mechanism for both survival and progression of cancer (Brock et al., 2009; Marusyk et al., 2012). Recent advances in single-cell sequencing provide powerful tools to explore genetic and functional heterogeneity, which have provided novel mechanisms in our understanding of carcinogenesis and revealed strategies for treatment (Puram et al., 2017; Lambrechts et al., 2018; Yost et al., 2019; Zhang et al., 2020).

Recent scRNA-seq studies provide novel perspectives that are helpful in advancing our understanding of OC progression (Shih et al., 2018; Geistlinger et al., 2020; Hu et al., 2020; Izar et al., 2020). However, effective diagnostic/therapeutic strategies remain indistinct, and the mechanisms associated with recurrence or metastasis are still poorly understood. A thorough exploration of OC relapse or metastasis could strengthen our understanding of the mechanisms associated with tumor carcinogenesis and progression and thus helpful for discovering more effective therapeutic strategies for OC.

In the present study, we thoroughly examine eight OC patients, namely, four with primary carcinoma, two with metastatic carcinoma, and two with recurrent carcinoma, with single-cell transcription. Here, we depict single-cell atlas of OCs and identify several diverse clusters. Significantly, we authenticate a subtype in epithelial cells consisting of more recurrent cells, which are more important across multiple clusters. Furthermore, we explore the clinical application of novel genes of this subtype with public datasets. In addition, interferon alpha inducible protein 6 (IFI6) is identified as a pivotal gene in OC progression. Notably, a variety of bioinformatic methods and experimental assays are conducted, revealing that IFI6 accelerates cell proliferation and influences cisplatin resistance. The potential mechanism may be involved in the NF-κB pathway. Last, we develop a prognostic model, RiskScore, which can be used as an independent prognostic factor in OC. Our work will be helpful to elucidate the biology of OC based on single-cell RNA-sequencing, thus providing clinical guidance in prognosis and treatment for OC patients.

## Materials and Methods

### OC and Other Cancer Datasets

Single-cell RNA-seq for OCs is extracted from GSE130000. Bulk RNA-seq data and relevant clinical data for TCGA cancers are obtained from UCSC Xena^1^. Multiple OC datasets downloaded from GEO are integrated with the sva package (Leek et al., 2012). All public datasets used in this study are described in Supplementary Table 1.

### Single-Cell RNA-Seq Data Preprocessing

The matrices for all samples are combined and processed with Seurat v3 (Butler et al., 2018). All functions are run with default parameters, unless otherwise specified. Low-quality cells (<300 genes/cell, <3 cells/gene, and >20% mitochondrial genes) are removed. The remaining cells are normalized by log-transformation. We select the top 2,000 highly variable genes (HGVs) to aggregate samples into a merged dataset and then scale. The batch effects among patients are eliminated with the harmony package (Korsunsky et al., 2019). The top 20 principal components, along with HGVs, are used in this process. The FindClusters function of the Seurat package is used for data clustering. The main cell clusters are visualized using the t-distributed stochastic neighbor embedding (tSNE) function. For sub-clustering analysis, we apply the same procedure of finding HGVs, dimensionality reduction, and clustering. The FindAllMarkers function is used to list markers of all clusters. We characterize the identities of cell types based on the CellMarker database (Zhang et al., 2019).

### Assessment of Tumor, Stromal, and Immune Score

The ESTIMATE algorithm is used to infer tumor purity, immune, and stromal score for each single cell with the ESTIMATE package (Yoshihara et al., 2013).

### The Chromosomal Copy Number Variation Estimation

Initial copy number variations (CNVs) for each region are estimated by the infer-CNV package (Patel et al., 2014). Non-epithelial cells are used as the reference. The CNVs of all cells are calculated by expression levels from single-cell sequencing data for each cell with a cutoff of 0.1.

### SCENIC Analysis

Transcription factor (TF) activity is analyzed using pySCENIC, a Python-based computational analysis tool of the SCENIC pipeline (Aibar et al., 2017). TF activity (AUC) for each cell is calculated with motif collection version mc9nr.

### Gene Set Functional Analysis

Predominantly, pathway analyses are conducted to evaluate the activation of hallmark pathways and metabolic pathways, which are summarized with the molecular signature database (Subramanian et al., 2005) and curated dataset (Gaude and Frezza, 2016), respectively. Then, we apply AUCell or GSVA package to estimate the pathway activity of each cell.

### Cell–Cell Communication Analysis

To explore the potential relationship between different subtypes of the epithelium and other cell types, a Python-based analysis tool, CellPhoneDB (Vento-Tormo et al., 2018), is used to analyze cell–cell communication at the molecular level and calculate ligand–receptor pairs for clusters. Cytoscape is used to visualize the network of clusters.

### Developmental Trajectory Inference

We use Monocle2 (Trapnell et al., 2014) to analyze the sample trajectories and explore the differentiation process of two clinical clusters. Differentially expressed genes across cluster transitions are calculated by the “differentialGeneTest” function. “DDRTree” is applied to reduce dimensions and “plot_cell_trajectory” is used for visualization.

### Deconvolution of Bulk Expression Data

The reference signature matrix is generated by the top 50 genes of each cell type (seven epithelial subtypes and other four clusters). The reference matrix and the TCGA OC dataset are deconvoluted by CIBERSORT, which is based on the non-parameter support vector regression and is robust to the interfering effects of noise and outliers (Newman et al., 2015). CIBERSORT is run in the relative mode with 1,000 permutations. Deconvolution analysis generates scores of 11 cell signatures in each tumor sample. This signature score could be interpreted as the relative abundance of the corresponding cell state in a particular tumor sample. Then, Cox regression and Kaplan–Meier survival curves are used to explore the survival of cell types.

### Public Data Analysis

To identify gene patterns of S1 and classify patients for further analysis, firstly, 18 of 344 genes of S1 are selected at *p* < 0.05 with the univariate Cox regression model in the TCGA OC dataset (Supplementary Table 2). Then, we employ the R package ConsensusClusterPlus, a consensus clustering algorithm (pam), to determine the optimal cluster number. TCGA OC patients are divided into two subgroups associated with the highest stability and the lowest ambiguity, which is validated by the GDSC dataset (Lu et al., 2019). Subsequently, Kaplan–Meier analysis is used to assess the survival of the two clusters with the TCGA OC dataset, and R package pRRophetic is used to estimate the IC_50_ for cisplatin and docetaxel in different clusters. To explore the important genes between the two clusters that exhibit different responses to treatment, we apply the random forest classification algorithm with the R package randomForest, which ranks the importance of genes with Gini values. The top five genes are IFI27, IFI6, TMEM258, COX7A2, and NDUFC2.

To explore the clinical application of IFI6, we built a novel RiskScore including IFI6 and five other genes. Firstly, 18 prognostic genes are selected at *p* < 0.05 with the univariate Cox regression model and then these genes are narrowed down using the lasso algorithm. The TCGA OC dataset is used as the training cohort and the GEO OC meta-dataset is deemed as the testing cohort (an integrated OC cohort: GSE18520, GSE19829, GSE26193, GSE30161, GSE63885, and GSE9891 with GPL570, Supplementary Table 1). Using OS as the predictive index, this procedure is repeated 10,000 times to construct the RiskScore. Last, the RiskScore is generated with gene expression values and corresponding lasso coefficients using the following formula:

Y=[CCDC34×(-0.157)+NDUFC2×(-0.206)+HMGN5×(-0.116)+SPEN×0.131+CLTA×(-0.225)+IFI6×0.19].

Kaplan–Meier survival analysis and time-dependent ROC curves are used to evaluate the performance of RiskScore. Patients are divided into a high- and low-RiskScore group based on the median value of RiskScore.

### Cell Culture and siRNA Transfection

In the present research, OC cell lines (including HEY, SKOV3, A2780, and CAOV8) are obtained from ATCC. HEY, SKOV3, and A2780 are cultured in Roswell Park Memorial Institute (RPMI)-1640 medium supplemented with 10% fetal bovine serum (FBS) and 100 U/ml penicillin/streptomycin at 37°C with 5% CO_2_. CAOV8 is cultured in high-glucose Dulbecco’s modified Eagle’s medium (DMEM) containing 10% FBS and 100 U/ml penicillin/streptomycin under similar conditions. All cell lines are transfected with Lipofectamine RNAmax. IFI6-target specific small interfering RNA (siRNA) is synthesized by JTSBIO Co., Ltd., (Wuhan, China). The sequences of IFI6-target-specifc-siRNA (siIFI6) are as follows: siRNA1, 5′-GCUGCUC UUCACUUGCAGUTTACUGCAAGUGAAGAGCAGCTT-3′; siRNA2, 5′-GCAGCGUCGUCAUAGGUAATTUUACCUAUGA CGACGCUGCTT-3′, siRNA3, 5′-CCACAAGUAUCUCGAUAG UTTACUAUCGAGAUACUUGUGGTT-3′; and the sequence of control is 5′-UUCUCCGAACGUGUCACGUTACGUGA TCACGUUCGGAGAATT-3′. All cells are cultured in six-well plates. Cells are transfected with 100 nmol/L siIFI6 or siCon and incubated for 24 h for subsequent assays.

### Cell Proliferation Assay

CCK-8 assay and plate clone formation assay are used to evaluate cell proliferation. CCK-8 assay is performed according to our previous study (Zhao et al., 2020). After transfecting siRNA for 24 h, A2780 cells are cultured in six-well culture plates (1,000/well) for 7 days. The cells are fixed with 4% paraformaldehyde (PFA) and stained with 0.1% crystal violet for 15 min and then photographed.

### RNA Extraction and RT-qPCR

RNA extraction and RT-qPCR are conducted according to our previous study (Zhao et al., 2020). The primer sequences are listed in Supplementary Table 3.

### Apoptosis Assays by Flow Cytometry

SKOV3 cells (5 × 10^5^) are cultured in six-well plates for 24 h followed by cell transfections for 24 h. Subsequently, cells are exposed to 10 μg/ml cisplatin. After 24-h treatment, the cells are collected to determine apoptosis using Annexin-V-(FITC) and propidium iodide (PI) kit (BD Biosciences, San Jose, CA, United States). The double-stained cells are subsequently analyzed by the BD flow cytometer.

## Results

### A Single-Cell Atlas of OCs

**FIGURE 1:**
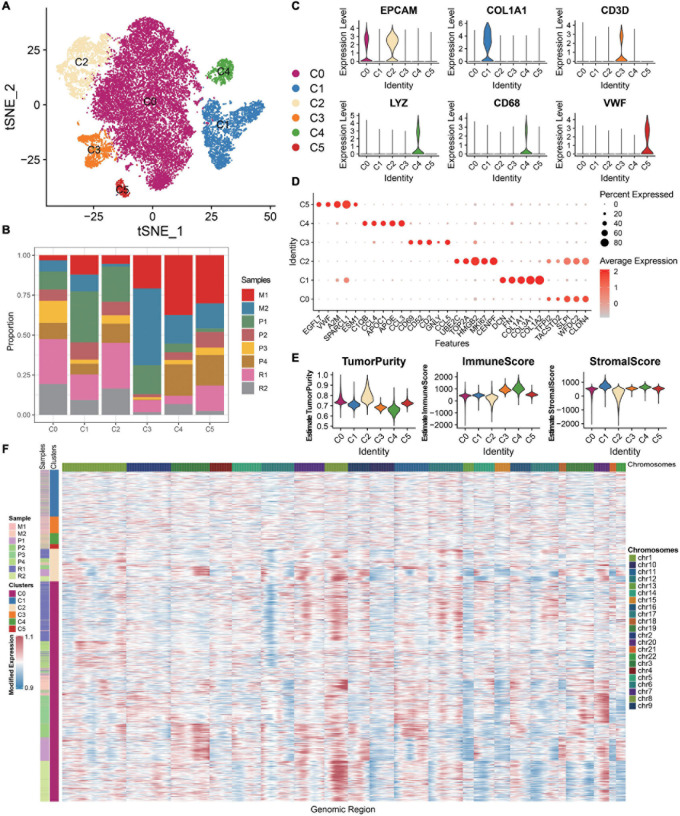
ScRNA-seq profiling of the primary, metastatic, and relapsed OCs. **(A)** tSNE plot annotating six major cell types in OCs. **(B)** Histogram indicating the proportion of diverse cell types across different sample origins. **(C)** Violin plots exhibiting the expression of representative markers across diverse cell types. The *y*-axis was the normalized read count. **(D)** Dot plot showing the expression of the top five markers in each indicated cell types. **(E)** Violin plots illustrating the estimation of tumor purity, immune score, and stromal score across different cell types. **(F)** Chromosomal landscape of inferred large-scale CNVs distinguishing malignant epithelial cells from other cells.

A total of eight samples, namely, four primary carcinomas, two metastatic carcinomas, and two recurrent carcinomas, are discussed. After removing low-quality cells, a total of 21,212 cells are finally acquired, namely, 3,242 cells from P1, 1,571 cells from P2, 2,186 cells from P3, 2,085 cells from P4, 1,489 cells from M1, 2,026 cells from M2, 5,191 cells from R1, and 3,422 cells from R2 (Supplementary Table 4). The single-cell number in our study that is different from GSE130000 may result from different filtration criteria. Considering the batch effects in different samples, we use the R package harmony to integrate these samples to eliminate the batch effects (Supplementary Figure 1). These cells are classified into six main cell lineages, namely, C0–C5 (Figure 1A). The corresponding proportion for each cluster is discrepant (Figure 1B). C0 and C2 are composed of a large proportion of recurrent carcinomas; C3 and C4 have a relatively higher proportion of metastatic tumors. Then, we annotate the cell clusters based on the average expression of the top five markers and well-known markers for each cluster (Figures 1C,D). We find that the atlas mainly comprises two epithelial cell types (i.e., C0 and C2), a mesenchyme clusters (C1), a T cell cluster (C3), a macrophage cluster (C4), and an endothelium cluster (C5). Then, we estimate tumor purity, immune, and stromal score for each single cell. Therefore, immune cells (T cell and macrophage cell) play important roles in OC metastasis. As shown in Figure 1E, mesenchyme cells (C1) show a higher stromal score, and immune cells (C3 and C4) exhibit a higher immune score. Cells scored low for both stromal and immune gene expression present higher tumor purity (C0 and C2) and express high levels of epithelial markers (EPCAM and WFDC2), promoting that these cells are malignant.

The chromosomal CNV score of each cell helps to identify the malignant clusters. We demonstrate that the epithelial cells (C0 and C2) exhibit remarkably higher CNV levels than other cell types across intervals of the genome (Figure 1F). Thus, C0 and C2 are malignant epithelial cells, supporting the fact that OC mainly originated from the epithelium. Studying the epithelial cells may help to understand the recurrence of OC.

### S1 Occupies a Dominant Role in OC Cells and Is Associated With Survival

**FIGURE 2:**
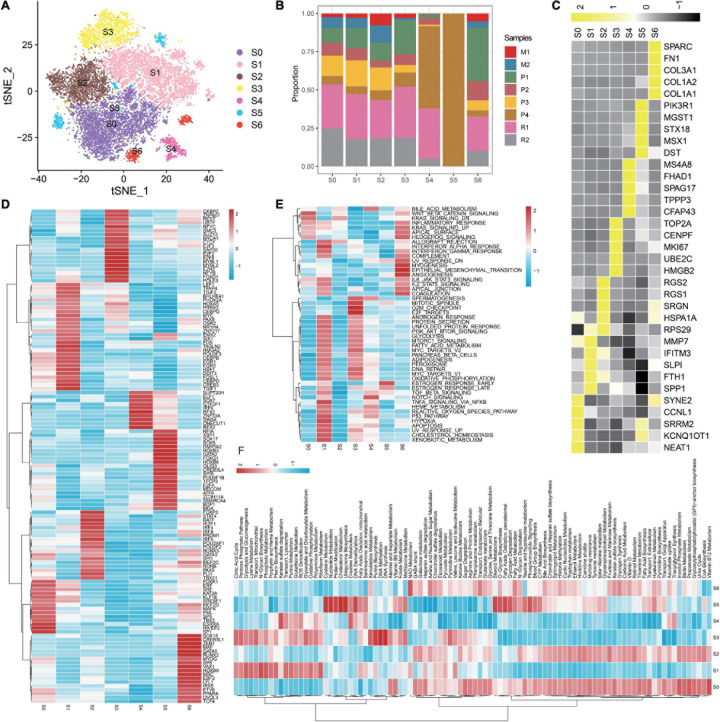
Detailed characterization of malignant epithelial cells. **(A)** tSNE plot showing seven subgroups generated from malignant epithelial cells. **(B)** Heatmap showing the average expression of the top five markers across different subgroups. **(C)** The proportion of diverse subgroups across different sample origins. Differences in the activities of the TFs **(D)**, HALLMARK pathway **(E)**, and metabolic pathways **(F)** in each malignant epithelial subgroup.

Overall, 16,146 malignant cells from eight tumor samples are identified and retained for further analyses. In this study, seven diverse subgroups are identified in the malignant epithelial cluster on the basis of the tSNE graph (Figure 2A), demonstrating the heterogeneity of epithelial cells in OC. Conspicuously, S0–S3 are composed of a larger proportion of recurrent carcinomas; S5 is almost composed of primary carcinomas (Figure 2B). We notice that specific markers of S1 are related to immunity such as SPP1, SLPI, and IFITM3 (Figure 2C). We then apply SCENIC analysis to explore TFs with gene expression differences across cell types (Figure 2D). As a result, a set of TFs related to carcinogenesis are enriched in S1, such as PAX8 and MYC. We further explore the functions of different epithelial subtypes by comparing pathway activities. As shown in the heatmap (Figure 2E), S1 enriches some hallmark terms related to immune, such as INTERFERON_ALPHA_RESPONSE, INTERFERON_GAMMA_RESPONSE, and TNFA_SIGNALING_VIA_NFKB. Notably, some metabolic terms are also enriched in S1 (Figures 2E,F), such as XENOBIOTIC_METABOLISM, Pterin biosynthesis, and Riboflavin Metabolism. Thus, S1 is a malignant epithelial and related to immunity and metabolism; discussing this subtype may help to understand the biological mechanisms of OC progression and seek for novel therapeutic targets.

**FIGURE 3:**
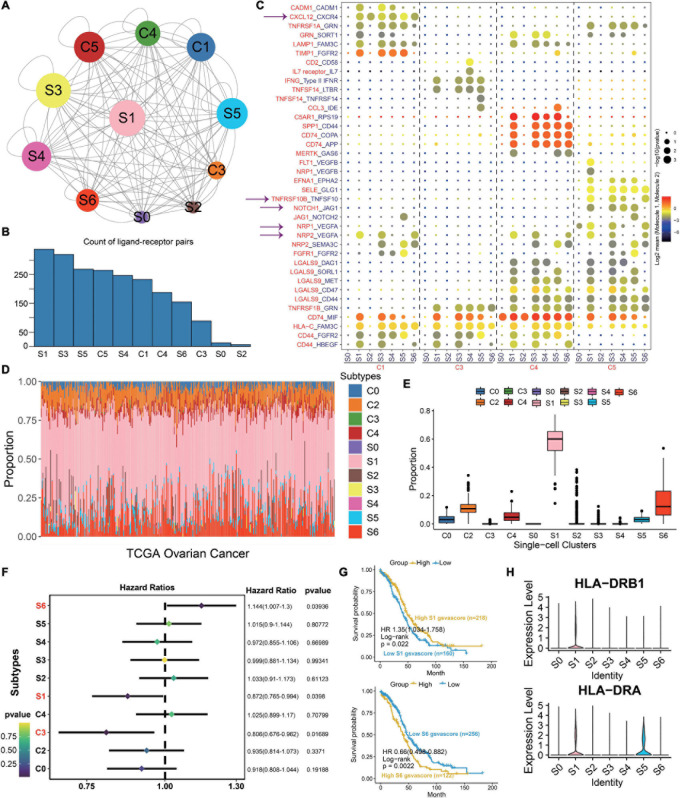
S1 is the dominant subtype in malignant epithelial subgroups. **(A)** Interaction network constructed by CellPhoneDB; circle size represents interaction counts. **(B)** Bar plot illustrating the count of ligand–receptor pairs. **(C)** Dot plot showing ligand–receptor pairs of malignant epithelial subgroups and other clusters. **(D)** Stacked bar plot summarizing cell subtype fractions with the deconvolution result of 379 OCs from TCGA. Colors of the bars represent 11 cell states as shown in the legend. The *y*-axis represents the proportion of each state in a tumor sample. In the *x*-axis, each column represents one tumor case. **(E)** Box plot illustrating the proportion of each state in TCGA OC samples. **(F)** Association between relative abundance of cell states calculated by CIBERSORT and survival. **(G)** Kaplan–Meier curves for the relative abundance of S1 and S6. **(H)** Violin plots exhibiting the MHC molecules such as HLA-DRB1 and HLA-DRA across different subtypes. The *y*-axis was the normalized read count.

To investigate the interaction network of epithelial subtypes and other clusters in OC, we utilize CellphoneDB to calculate potential ligand–receptor pairs. Notably, S1 shows the most interaction pairs with other cell types (Figures 3A,B), revealing its dominant role in OC. As shown in Figure 3C, CXCR4, TNFSF10, VEGFA, and JAG1 secreted by S1 interact with receptors expressed on mesenchyme, immune cells, and endothelium cells. These ligand–receptor pairs may be related to immune, angiogenesis, and CAF proliferation.

Then, we use the deconvolution method to explore the proportion of each cell type with the TCGA OC dataset. The cell-type proportions are shown in Figure 3D, and box plots depict their distributions (Figure 3E). Notably, S1 exhibits the highest proportion across all cell types, followed by S6. To explore the importance of clusters, we employ the Cox regression to discuss the prognosis of each cluster. As a result, S1, S6, and C3 are statistically significant in OC (*p* < 0.05, Figure 3F). The HR and 95% CI for S1 is 0.872 (0.765–0.994), which for S6 and C3 are 1.144 (1.007–1.3) and 0.806 (0.676–0.962), respectively. Kaplan–Meier curves also show that S1 and S6 are associated with survival (*p* < 0.05, Figure 3G). Above all, we deduce that S1 plays an important role in OC carcinogenesis. Moreover, MHC molecules such as HLA-DRB1 and HLA-DRA highly express in S1 (Figure 3H), consistent with our previous results that S1 is closely related with immunity.

### Association of Markers in S1 With Clinical Treatment Subtypes

**FIGURE 4:**
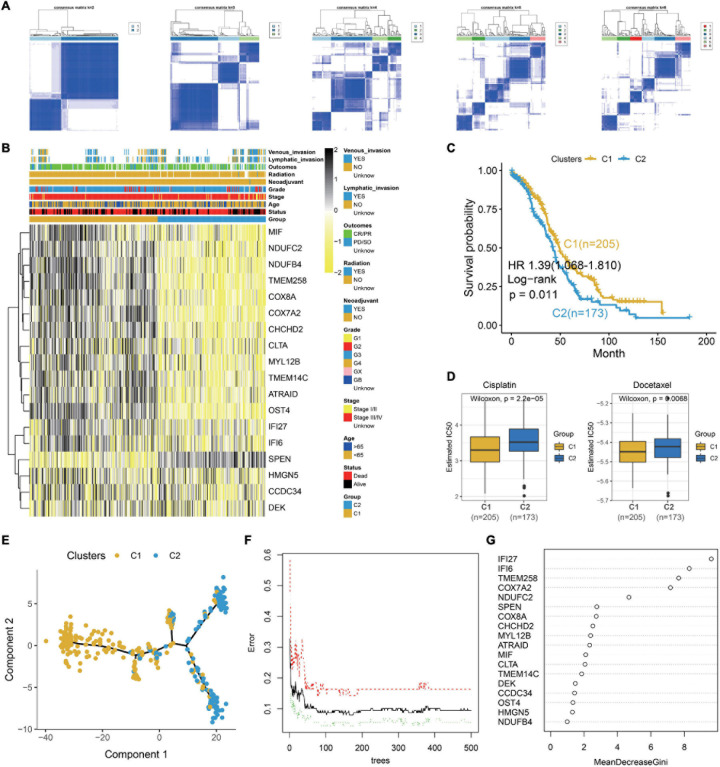
Identification of two clinical treatment subtypes from the TCGA OC dataset. **(A)** Consensus matrixes of the TCGA OC cohort for *k* = 2–6. **(B)** Heatmap of 18 genes in the TCGA OC dataset. **(C)** Kaplan–Meier plot for two clusters in the TCGA OC dataset. **(D)** Box plot illustrating that higher IC_50_ for cisplatin and docetaxel in C2. **(E)** Pseudotime graph demonstrating the differentiation process from C1 to C2. **(F)** Error rate for the data as a function of the classification tree. **(G)** Importance of 18 genes for the predictors.

To explore the clinical application of gene expression patterns in S1, we use univariate Cox regression to narrow down 344 markers. As a result, 18 genes are associated with survival and selected at *p* < 0.05. Then, 379 OC patients are divided into two different subtypes with ConsensusClusterPlus based on the 18 markers (Figures 4A,B). The relationship of the markers is illustrated in Supplementary Figure 2. Compared to the patients from C1, patients in C2 show worse outcome (Figure 4C). Cisplatin and docetaxel are classical treatment in OCs. Interestingly, we discover that IC_50_ for cisplatin and docetaxel is higher in C2 (Figure 4D), meaning that these patients are drug resistant. The above results are validated with the GDSC dataset (Supplementary Figure 3), demonstrating that our classification is stable and robust. Moreover, pseudotime graph illustrates a differentiation process from C1 to C2, confirming heterogeneity between the two clusters (Figure 4E).

Then, we explore the functions of the two clusters which reveal different survival and drug responses. Meaningfully, C1 enriches in immune and C2 is concerned with ECM and drug metabolism (Supplementary Figure 4). To further explore the pivotal gene that alters the functional state and drug reaction between the two clusters, the 18 prognostic markers are subjected to random forest algorithm. As a result, IFI27, IFI6, TMEM258, COX7A2, and NDUFC2 are the top five important genes in our clusters (Figures 4F,G). We explore their expression in different epithelial subtypes, finding that IFI6 is the unique gene that specifically expresses in S1 (Supplementary Figure 5). In our next work, we explore the carcinogenesis of IFI6 in OC.

### Downregulation of IFI6 Suppresses Proliferation and Potentiates Sensibility to Cisplatin Treatment of OC Cells

**FIGURE 5:**
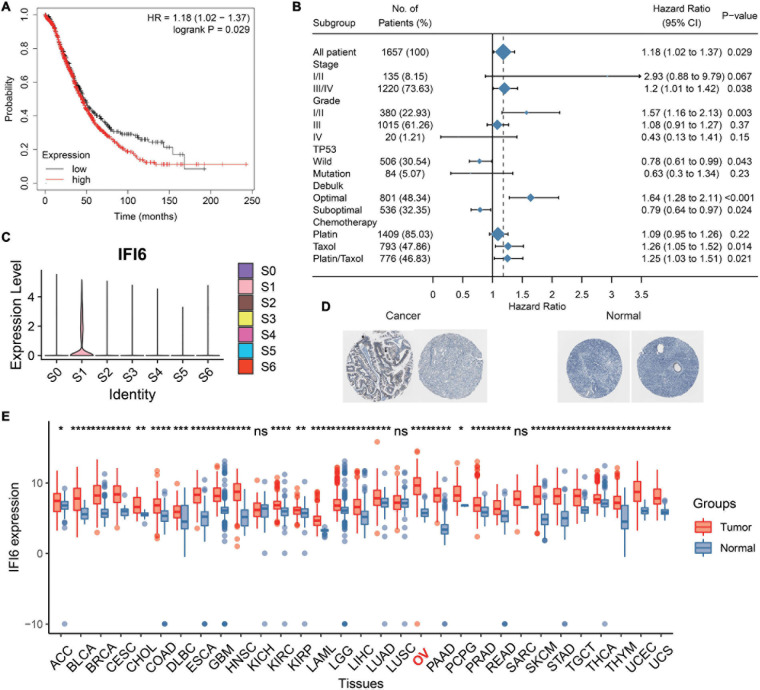
Analysis of prognosis and expression level of IFI6 in OC. **(A)** Kaplan–Meier curve illustrating higher IFI6 accompanied by poor OS. **(B)** Survival associated with IFI6 in different subgroups of OC patients. **(C)** IFI6 has a high expression in S1. **(D)** Representative protein expression levels of IFI6 were high in OCs based on the Human Protein Atlas database. **(E)** Box plot showing higher IFI6 in OCs than paired normal samples.

IFI6 is reported to be carcinogenic in cancers (Gupta et al., 2016; Cheriyath et al., 2018; Liu et al., 2020). Thus, we deduce that IFI6 plays an important role in OC progression. We extract the survival data of IFI6 in 1,657 OC patients using Kaplan--Meier plotter^2^. IFI6 upregulation is found to be associated with poor OS in OC (Figure 5A and Supplementary Figure 6). Forest plot reveals the impact of IFI6 expression on patient outcomes in different clinical status. Although the results of subgroup analysis are heterogeneous, IFI6 is supported as a poor prognostic biomarker in most subtypes (Figure 5B). Overall, patients with higher IFI6 are accompanied with poor overall survival. In the stage III/IV group, patients with higher IFI6 present as poor survival with significance. In the stage I/II group, higher IFI6 is also associated with poor survival, but it is not statistically significant. Stratified by tumor grades, we find that higher IFI6 is associated with poor survival with significance in the grade I/II group. Upon stratification of the samples according to TP53 mutation status, significant differences in survival were observed between low- and high-IFI6 groups in the TP53 wild group. In the chemotherapy analysis, higher IFI6 is related to poor survival in patients treated with taxol, supporting the idea that they may be resistant to taxol. In truth, IFI6 is the unique gene that specifically expresses in S1 (Figure 5C and Supplementary Figure 5). The mRNA expression level of IFI6 is higher in multiple cancers including OC than paired normal samples in GTEx (Figure 5E). Noteworthy is the observation that the representative protein expression level of IFI6 is positive in OCs based on the Human Protein Atlas (HPA) database (Figure 5D). Briefly, a total of 11 OC patients including one moderate and 10 weak are positive considering the intensity of each IHC. As compared, the intensity of three normal ovary samples is negative.

**FIGURE 6:**
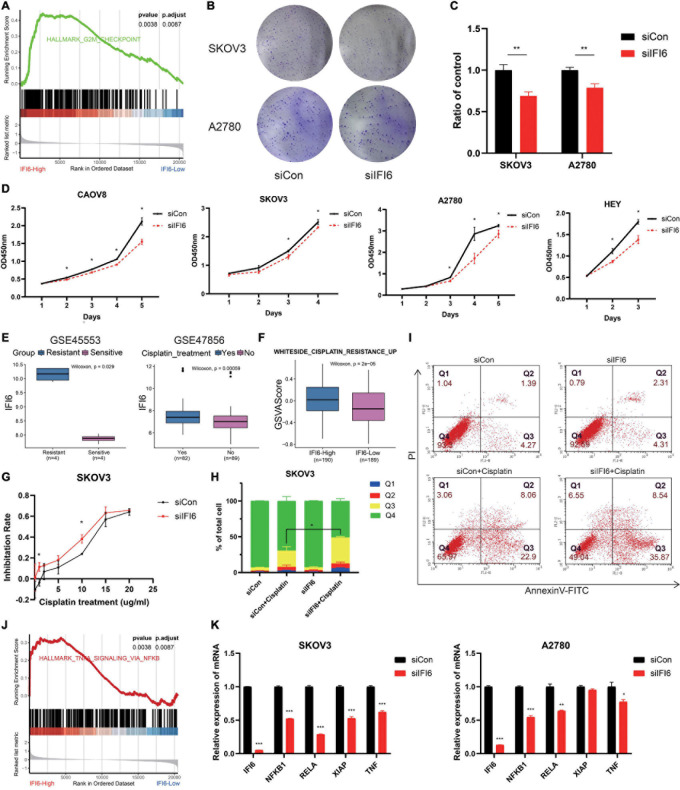
Potential mechanism of IFI6 promoting OC proliferation and cisplatin resistance may be related to the NF-κB pathway. **(A)** GSEA showing high-IFI6 groups is associated with G2M checkpoint in the TCGA OC dataset. **(B,C)** Effect of IFI6 on the colony formation of OC cells by plate colony formation assay. **(D)** CCK-8 assays demonstrating slower proliferation in IFI6 silent cells compared to the siCon cells. **(E)** Box plot illustrating higher IFI6 in the cisplatin-resistant and relapse group based on GEO datasets. **(F)** GSVA of WHITESIDE_CISPLATIN_RESISTANCE_UP in high- and low-IFI6 groups. **(G)** Cisplatin sensitivity in siCon and siIFI6 in SKOV3 cells. **(H,I)** Apoptotic cells by flow cytometry in control and siIFI6 in SKOV3 untreated or treated with cisplatin. The *p*-value of apoptosis (Q3) between the groups siCon+Cisplatin and siIFI6+Cisplatin is <0.05. **(J)** GSEA presenting higher IFI6 is associated with the NF-κB pathway. **(K)** Compared with control cells, the expressions of NFKB1, RELA, XIAP, and TNF are lower in siIFI6 cells in mRNA level.

After analyzing the public data, we sought to determine whether targeting IFI6 expression in OC cells could be used as a practicable therapeutic target to inhibit cell viability. In order to investigate the function of IFI6 in OC, we divide people into two groups according to the median of IFI6 in TCGA OC dataset. GSEA shows that high-IFI6 group is mainly associated with G2M checkpoint (Figure 6A), demonstrating that IFI6 is associated with cell cycle progression and accelerates cell proliferation. We further investigate the effects of siIFI6 on cell proliferation with CCK-8 assay and plate clone formation assay. As a result, downregulation of IFI6 decreases the proliferation of OC cell lines (Figures 6B–D).

Cisplatin is an important therapeutic drug in OC; however, its therapeutic effect is impeded by drug resistance. Exploring new targets and explaining resistance mechanisms are urgent and vital. In the present study, we find that IFI6 is higher in the cisplatin resistance and relapse group (Figure 6E). WHITESIDE_CISPLATIN_RESISTANCE_UP is more enriched in the IFI6-High group with GSVA algorithm (Figure 6F). These results prompt that IFI6 is associated with cisplatin resistance. In order to discuss the effect of IFI6 on the sensitivity of OC cell lines to cisplatin, CCK-8 assay and flow cytometry are performed. Forty-eight hours after transfection, SKOV3 cells are treated with increasing concentrations of cisplatin for 24 h, and their inhibition rate is measured by CCK8. As expected, SKOV3 cells transferred with siIFI6 are more sensitive to cisplatin toxicity (Figure 6G). Furthermore, SKOV3 cells transferred with siIFI6 significantly aggrandize cisplatin-induced apoptosis when compared to the siCon cells (Figures 6H,I). This clearly proves concrete evidence for the role of IFI6 in drug resistance.

Furthermore, to determine the molecular mechanism of IFI6 in promoting proliferation and cisplatin resistance, GSEA is conducted, and the result illustrates that the high-IFI6 group is enriched in the NF-κB pathway (Figure 6J). Convincingly, IFI6 is also positive with markers of the NF-κB pathway in cancers (Supplementary Figure 7). Then, we conduct RT-qPCR to validate the result. Interestingly, mRNA levels of NFKB1, RELA, XIAP, and TNF are lower in the silent IFI6 group compared to the siCon group (Figure 6K). Thus, we infer that IFI6 promoting OC progression may be involved in the NF-κB pathway.

### Construction of a Robust Prognostic Model Associated With IFI6

**FIGURE 7:**
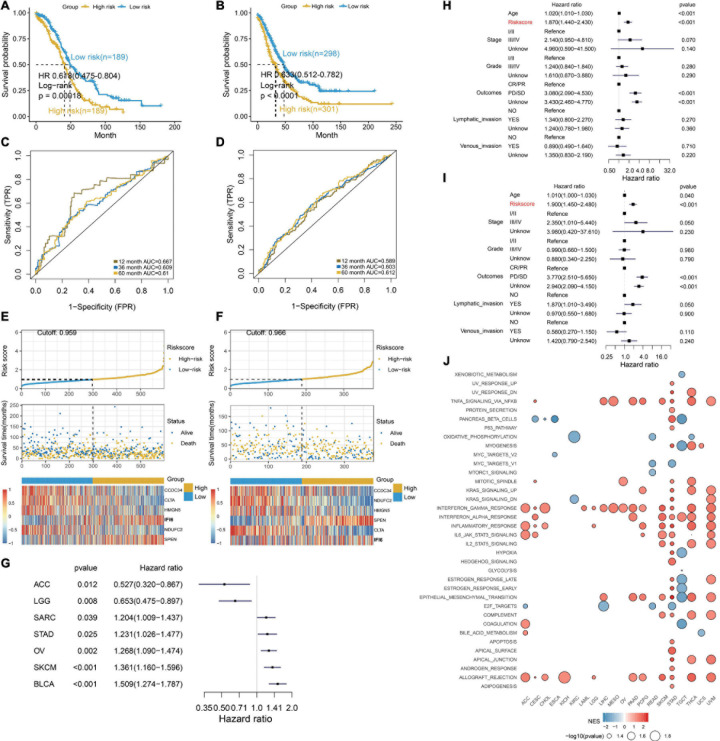
Excellent prognosis of the RiskScore. **(A,B)** Kaplan–Meier survival curves, **(C,D)** time-dependent ROC curve analyses, **(E,F)** RiskScore distribution, survival status, and gene expression profile for patients in high- and low-RiskScore groups in the TCGA OC cohort and GEO OC dataset. **(G)** Forest plot illustrating the OS of the RiskScore in multiple cancers. SARC, sarcoma; STAD, stomach cancer; OV, ovarian cancer; SKCM, Melanoma; BLCA, bladder cancer; ACC, adrenocortical cancer; LGG, lower grade glioma. **(H,I)** RiskScore is determined to be an independent and robust prognostic factor for OC samples by univariate Cox regression **(H)** and multivariable Cox regression **(I)** analysis. **(J)** Dot plot showing the functions of RiskScore across multiple cancers.

To deeply discuss the clinical application of IFI6, we use lasso algorithms to construct the RiskScore model including IFI6 and five other genes. The formula of the RiskScore is *Y* = [CCDC34 × (−0.157) + NDUFC2 × (−0.206) + HMGN5 × (−0.116) + SPEN × 0.131 + CLTA × (−0.225) + IFI6 × 0.19]. Patients with a lower RiskScore exhibit greater OS (log-rank *p* < 0.01; Figures 7A,B). Significantly, RiskScore is reliable to predict the survival of OCs based on time-dependent ROC curves. As a result, the area under curve (AUC) is 0.667, 0.609, and 0.661 in 1-year, 3-year, and 5-year survival, respectively (Figure 7C). In the validation dataset, the AUC is 0.589, 0.603, and 0.612 for 1-year, 3-year, and 5-year survival, respectively (Figure 7D). Calibration plots for RiskScore demonstrate that the model is reliable (Supplementary Figure 8). RiskScore analysis for six specific biomarkers in OC patients are shown in Figures 7E,F. Furthermore, the forest plot reveals diverse OS of RiskScore across multiple cancers in TCGA (Figure 7G). RiskScore is associated with poor survival in SARC, STAD, OV, SKCM, and BLCA. In contrast, RiskScore is associated with better survival in ACC and LGG. The RiskScore is determined to be an independent and robust prognostic factor for OC samples with univariate and multiple Cox regression analysis (Figures 7H,I). This indicates the good potential of RiskScore in survival monitoring. Moreover, we utilize GSEA to investigate the biological functions of RiskScore. The dot plot illustrates that RiskScore activates carcinogenesis-related terms and is associated with an inflammatory response (Figure 7J), such as EPITHELIAL_MESENCHYMAL_TRANSITION, COMPLEMENT, TNFA_SIGNALING_VIA_NFKB, INFLAM MATORY_RESPONSE, and IL2_STAT5_SIGNALING.

## Discussion

Despite the fact that many progresses in treatment strategies have improved overall survival rates, the clinical outcomes of OC remain depressed due to the high incidence of recurrence or metastasis, even after combination therapy (Miller et al., 2009). Treatment strategies of recurrent or metastasis tumors are often based on the primary tumor, although the molecular and pathological features are distinguishing. Studies have attempted to explore the mechanisms or therapeutic targets of metastasis or recurrent cancer (Lee et al., 2013; Samardzija et al., 2015). Exploration of a more effective treatment approach to improve patient outcome is important. However, identified markers or therapeutic targets with a complete disregard for intra-tumoral heterogeneity are not suitable for all patients. In recent years, mechanisms in cancer carcinogenesis and treatment strategies with scRNA-seq (Puram et al., 2017; Lambrechts et al., 2018; Yost et al., 2019; Zhang et al., 2020) in consideration of tumoral heterogeneity have been promoted and are more convincing. Thus, it is highly desirable to explore the underlying mechanisms between primary, metastasis, and recurrent OC, and then develop more effective therapeutic targets and prognostic biomarkers with scRNA-seq in OC patients.

In this study, we establish a comprehensive single-cell expression atlas and identify two malignant epithelial cell types composed of a large proportion of recurrent carcinomas. Thus, studying the epithelial cells may help to understand the recurrence of OC. Epithelial ovarian cancer (EOC) accounts for the majority OC cases and advanced EOC eventually develops into a recurrent platinum-resistant disease. These malignant epithelial cells are further explored and reclustered to seven subtypes. Each subtype shows distinct functions and markers, revealing substantial heterogeneity among OCs. Notably, multiple carcinogenesis and immune-related terms and markers are enriched in S1. Prognostic S1 exhibits the highest proportion in OC and has the most ligand–receptor pairs with other cells. Meaningfully, these interaction pairs are related to immune, angiogenesis, and CAF proliferation. The above reveals the dominant role of S1 in OC.

To explore the clinical application of S1, we classify patients into two clusters with 18 prognostic genes. Significantly, patients in C1 are associated with favorable survival and more sensitive to cisplatin and docetaxel. Furthermore, we find a differentiation process from C1 to C2. Then, we explore the functions of the two clusters and found that C1 is associated with immune and C2 is concerned with ECM and drug metabolism. This further confirms that the two distinct clusters may play different roles in OC progression and treatment. In the subsequent in-depth analysis, we identify IFI6 as an important gene in predicting the two clusters, which may be essential for carcinogenesis and therapeutic strategies in OC.

IFI6, an interferon (IFN)-stimulated gene (ISG), is mainly enriched in the inner mitochondrial membrane (Liu et al., 2020) and plays important roles in immune modulation (Liu et al., 2019). IFI6 is reported to overexpress in multiple malignant cancers (Gupta et al., 2016; Cheriyath et al., 2018; Liu et al., 2020) and is identified as a survival factor (Cheriyath et al., 2007; Liu et al., 2020). Upregulation of IFI6 may contribute to cancer progression; however, the roles of IFI6 in OC carcinogenesis remain unclear. In the current research, prognostic IFI6 is higher in ovarian tumors compared to the normal ovary in mRNA and protein levels. Although the understanding of its biological functions is limited, IFI6 is characterized as a proliferative factor (Gupta et al., 2016; Liu et al., 2020) and associated with metastasis (Cheriyath et al., 2018). Studies reveal that IFI6 influences proliferation *via* regulating DNA replication stress (Gupta et al., 2016) and mediating ROS accumulation (Liu et al., 2020). In the present study, GSEA is performed and demonstrates that IFI6 is related to G2M checkpoint. The G2/M DNA damage checkpoint prevents the cell from entering mitosis (M phase) if the genome is damaged; thus, the proliferation is inhibited. As validation, the *in vitro* assays disclose that IFI6 promotes cell proliferation. However, we do not have further information of IFI6 influencing cell proliferation by G2M checkpoint. In future work, we will explore the relationship of IFI6 and G2M checkpoint.

Cisplatin is one of the most commonly used drugs for OC treatment, and cisplatin cytotoxicity has been attributed to DNA binding, single-stranded DNA breaks, and further induction of cell death (Cepeda et al., 2007). However, cisplatin resistance is a clinical challenge for patient treatment (Pujade-Lauraine et al., 2019). IFI6 is reported to be associated with chemoimmunotherapy (Moschella et al., 2013) and tamoxifen resistance (Cheriyath et al., 2012). In the present study, we find that higher IFI6 is associated with cisplatin resistance. We also observe that depletion of IFI6 significantly reveals a higher inhibition rate and reinforces cisplatin-induced apoptosis in OC cell lines, suggesting that IFI6 potentiates the effect of cisplatin sensitivity. However, the mechanisms of IFI6 influencing cisplatin resistance in OC remain unknown. Cisplatin resistance leads to therapeutic failure, and the mechanisms may be involved in enhanced DNA damage repair, imbalance of cisplatin uptake and efflux, and altered regulatory pathways (Galluzzi et al., 2012). It is reported that IFI6 is necessary for cancer progression *via* regulating DNA replication stress (Gupta et al., 2016), thereby inhibiting cell apoptosis and modulating cisplatin resistance. This needs to be further confirmed in our subsequent work. Exploring the effect of IFI6 on OC may help to understand the regulatory mechanisms of cisplatin-resistance generation, assisting therapeutic strategies in the clinical setting.

Recent studies have ascribed a prominent role of IFI6 for mitochondrial reactive oxygen species (mtROS) in metastasis (Porporato et al., 2014; Rivadeneira et al., 2015). Whether IFI6 promotes OC progression by another mechanism remains elusive. In our present study, mechanism analysis demonstrates that IFI6 is positively associated with the NF-κB pathway. As a validation, our experiments reveal that the levels of NFKB1, RELA, XIAP, and TNF are lower in the siIFI6 group. Mechanistically, NF-κB activity can be induced under stress conditions, including DNA damage (Zhang et al., 2018). Moreover, NF-κB activation slows down the cell cycle, inducing anti-apoptotic proteins and stemness, thereby conferring pro-tumorigenic and resistance to chemotherapy (Zhang et al., 2018; Yang et al., 2020; Kumar et al., 2021). Thus, we infer that IFI6 may affect the activity of the NF-κB pathway, leading to pro-tumorigenesis and cisplatin resistance. With respect to tumorigenesis, NF-κB signaling is also a master regulator of the inflammatory response and increases pro-tumorigenic inflammation (Li et al., 2019; Ni et al., 2020; Gu et al., 2021). IFI6 may influence the inflammatory response by interacting with the NF-κB pathway, thus modulating OC progression.

IFI6 has an effect on proliferation and cisplatin resistance, and we further develop a RiskScore including IFI6 to explore the clinical application in prognosis. Exhilaratingly, RiskScore is an independent prognostic factor and presents encouraging prognostic value in predicting survival in OC and other cancers. Then, GSEA is further conducted to clarify the mechanisms of RiskScore. The result hints that RiskScore may affect the survival of OCs *via* carcinogenic terms, such as EPITHELIAL_MESENCHYMAL_TRANSITION. RiskScore is also associated with immune-related terms, corresponding to functions of two previous clusters. During the past years, several signatures have been identified for prognostic prediction based on the bulk mRNA transcription dataset (Fan et al., 2020; Wu et al., 2020). In this study, RiskScore generated with carcinogenic genes in the single-cell level seems to be more credible. Our study comprehensively analyzes the single-cell RNA-sequencing of OC and provides novel ideas for predicting outcomes and therapeutic strategies in OCs.

## Conclusion

In conclusion, we authenticate S1 as the dominant cells in OC and identify IFI6 as the vital gene in OC carcinogenesis. We demonstrate a potential mechanism of IFI6, influencing carcinogenesis, cell proliferation, and cisplatin resistance, which may be involved in the NF-κB pathway in OC. Significantly, we develop a prognostic model, RiskScore, which is an independent prognostic factor and presents encouraging prognostic values. Thus, this approach is potentially helpful for personalized anti-cancer strategies and predicting outcomes in the setting of OC.

## Data Availability Statement

The original contributions presented in the study are included in the article/Supplementary Material, further inquiries can be directed to the corresponding author/s.

## Author Contributions

HZ and WY contributed to the conception and design of the study. HZ drafted the manuscript and prepared all figures and tables. WY revised the manuscript. ZL, YG, JL, and XZ provided help for the assays. All authors read and approved the final version of this manuscript.

## Conflict of Interest

The authors declare that the research was conducted in the absence of any commercial or financial relationships that could be construed as a potential conflict of interest.

## Publisher’s Note

All claims expressed in this article are solely those of the authors and do not necessarily represent those of their affiliated organizations, or those of the publisher, the editors and the reviewers. Any product that may be evaluated in this article, or claim that may be made by its manufacturer, is not guaranteed or endorsed by the publisher.

## Footnotes

1
https://xenabrowser.net/datapages/

2
http://kmplot.com/analysis/index.php?p=service&cancer=ovar
