# Supplementary material for: Single-Cell RNA-Sequencing Portraying Functional Diversity and Clinical Implications of IFI6 in Ovarian Cancer
Source: Front Cell Dev Biol. 2021 Aug 25;9:677697. doi: 10.3389/fcell.2021.677697 (PMC8425592; doi:10.3389/fcell.2021.677697)
Supplement: Supplementary file 1 [file Presentation_1.pdf]

Single-cell RNA-sequencing portraying functional diversity and clinical implications of IFI6 in ovarian cancer

Hongyu Zhao[1], Zhefeng Li[1], Yan Gao[1], Jie Li[1], Xiaoting Zhao[1#], Wentao Yue[1#]

1 Central Laboratory, Beijing Obstetrics and Gynecology Hospital Capital Medical University, Capital Medical University, Beijing 100026, China

[#] Corresponding authors.

E-mail: yuewt@ccmu.edu.cn (WTY), zhao_xiaoting@126.com (XTZ)

Fig. S1 tSNE plots color-coded for annotated OCs by unaligned (A) and aligned (B) clustering.

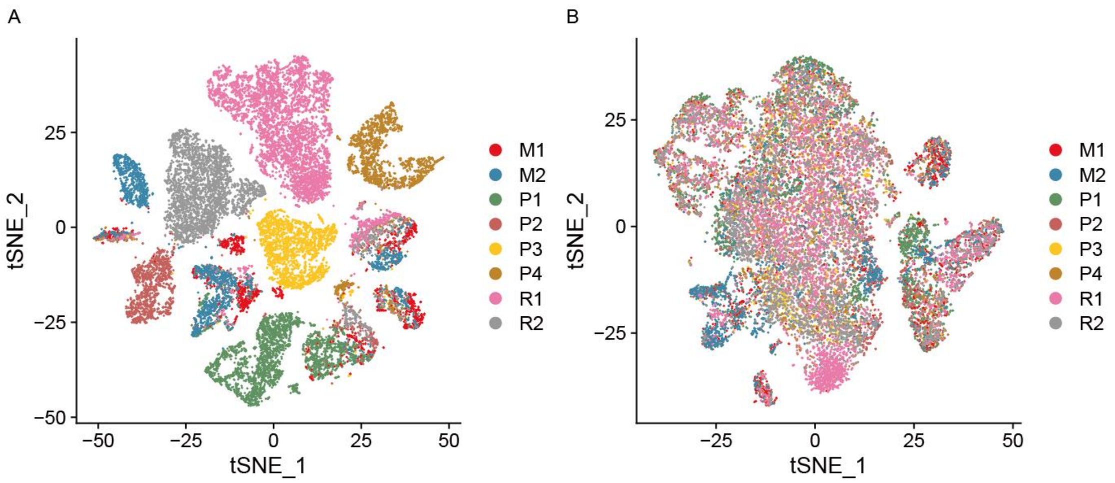

Fig. S2 Landscape of 18 gene interactions in the TCGA OC cohort.

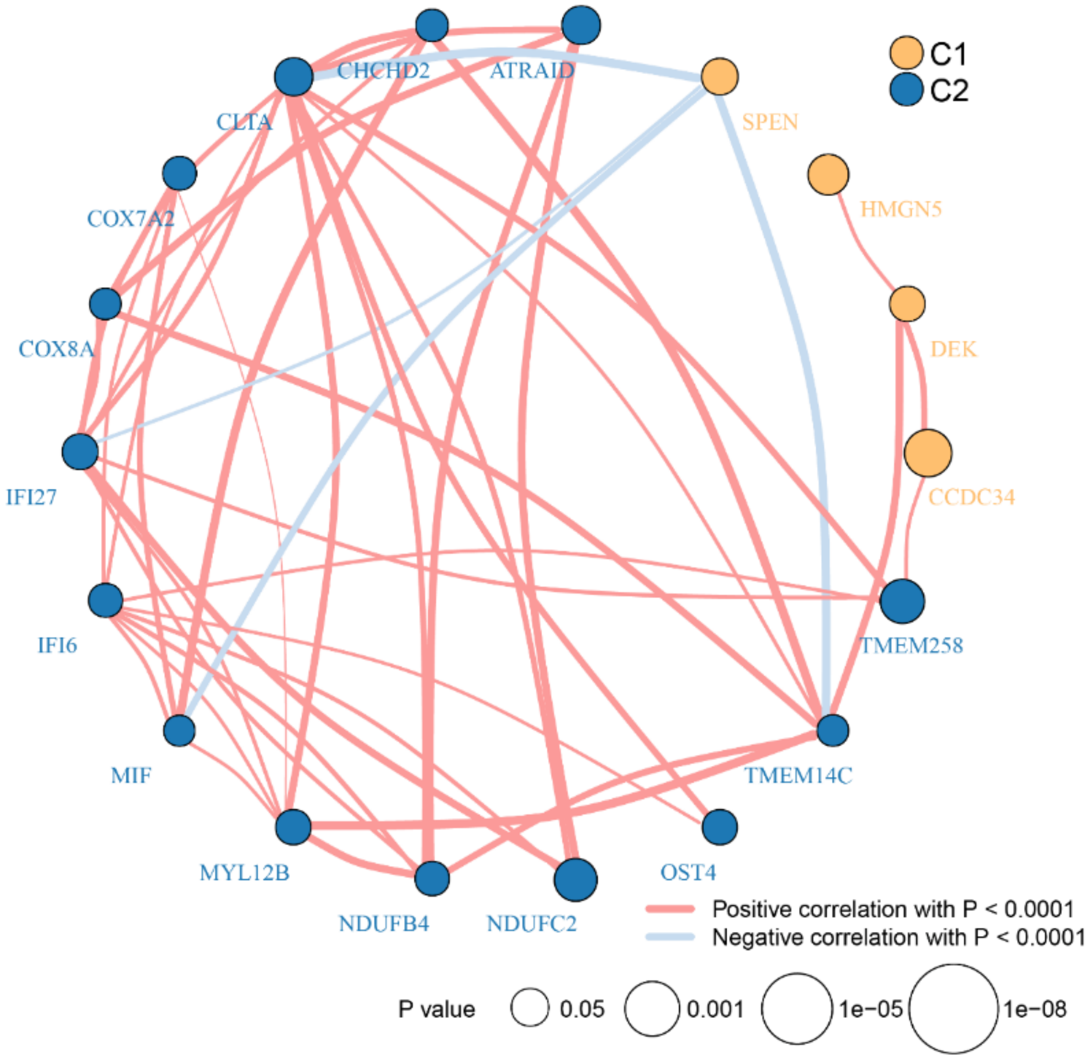

Fig. S3 Identification two clinical treatment subtype with GDSC dataset

(A) Consensus matrixes of GDSC for k = 2–6. (B) Heatmap of 18 gene in GDSC dataset. (C) Boxplot of IC50 for cisplatin and docetaxel between C1 and C2.

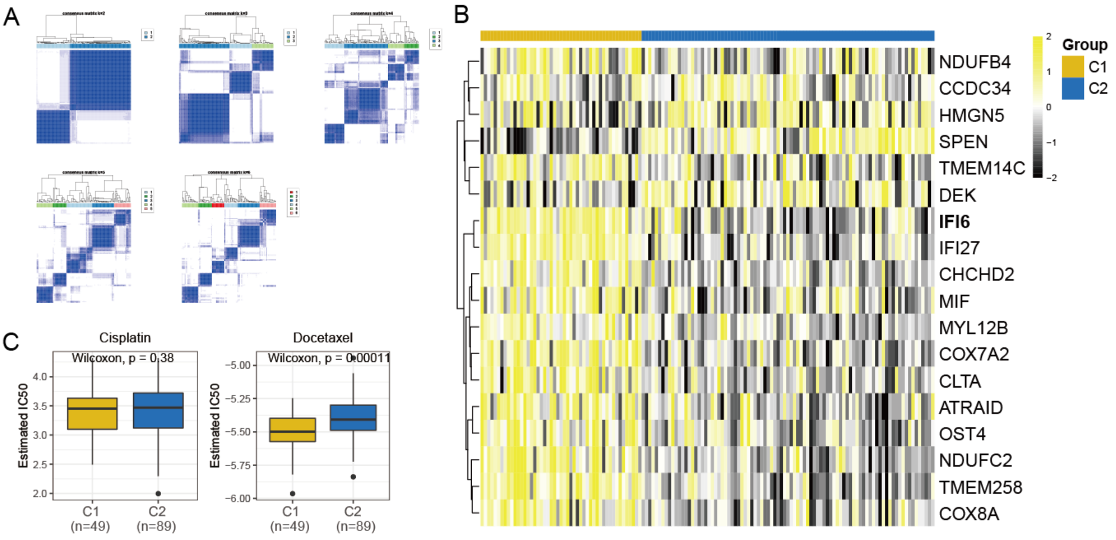

Fig. S4 Functional analysis of differential expression genes (DEGs) between C2 and C2.

(A)Volcano plot of DEGs between C2 and C1. GO (B) and KEGG (C) of C1. GO (D) and KEGG (E) of C2.

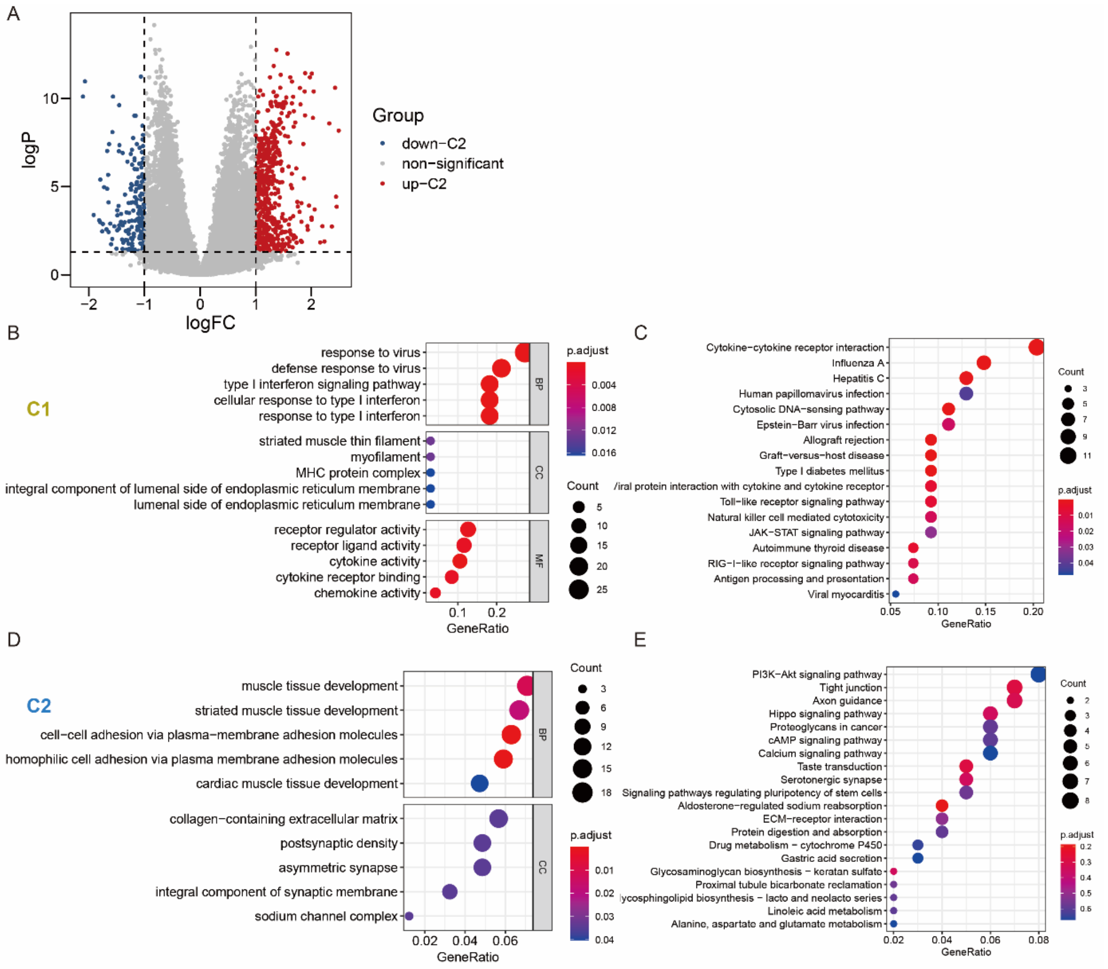

Fig. S5 Violin plots exhibiting the expression of representative markers across diverse cell types. The y axis was the normalized read count.

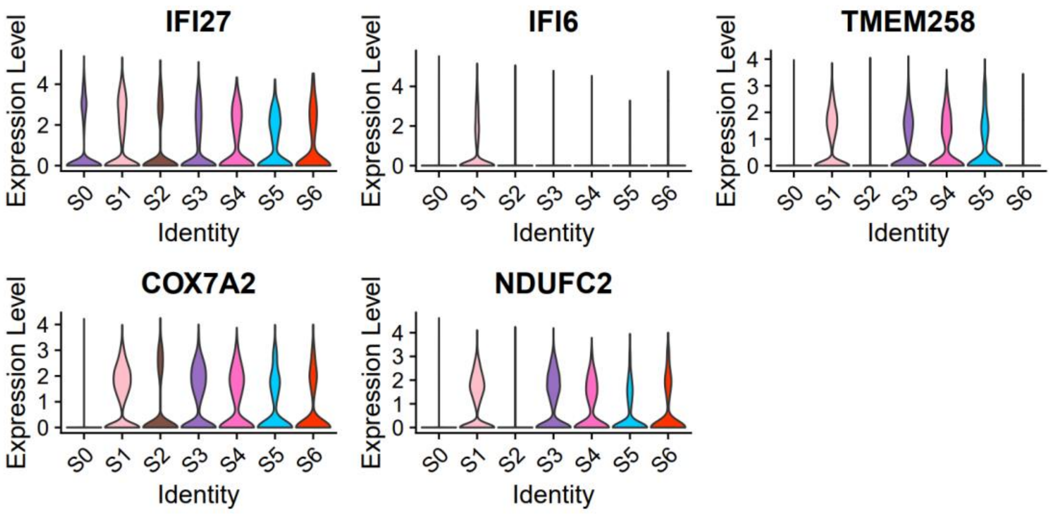

Fig. S6 Forest plot illustrating the survival associated with IFI6 in different datasets of OC patients.

GEO datasets	Hazard ratio	pvalue
GSE19829	0.200(0.060−0.660)	0.004
GSE15622	0.660(0.300−1.460)	0.300
GSE14764	0.700(0.290−1.670)	0.420
GSE18520	0.720(0.390−1.340)	0.300
GSE26712	0.780(0.550−1.120)	0.180
GSE3149	1.380(0.850−2.260)	0.190
GSE9891	1.420(0.980−2.080)	0.066
GSE26193	2.100(1.320−3.330)	0.001
GSE63885	2.110(1.220−3.620)	0.006
GSE30161	2.180(0.940−5.030)	0.063
GSE23554	4.510(1.500−13.590)	0.003
GSE27651	5.430(1.950−15.120)	<0.001

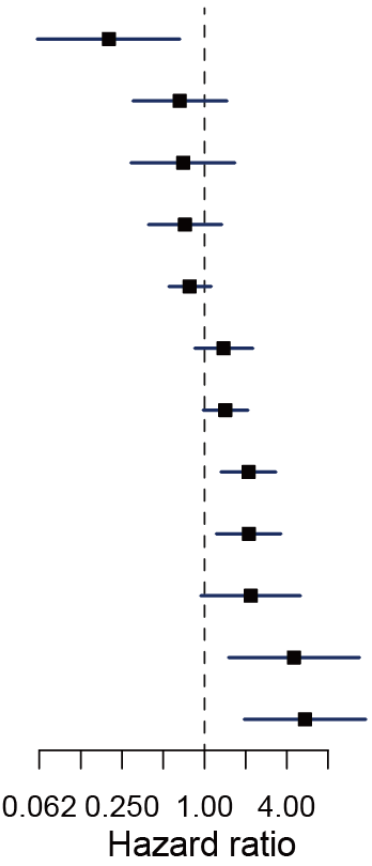

Fig. S7 Correlation of IFI6 and markers of NF-κB pathway based on TCGA, every dot represents one cancer type.

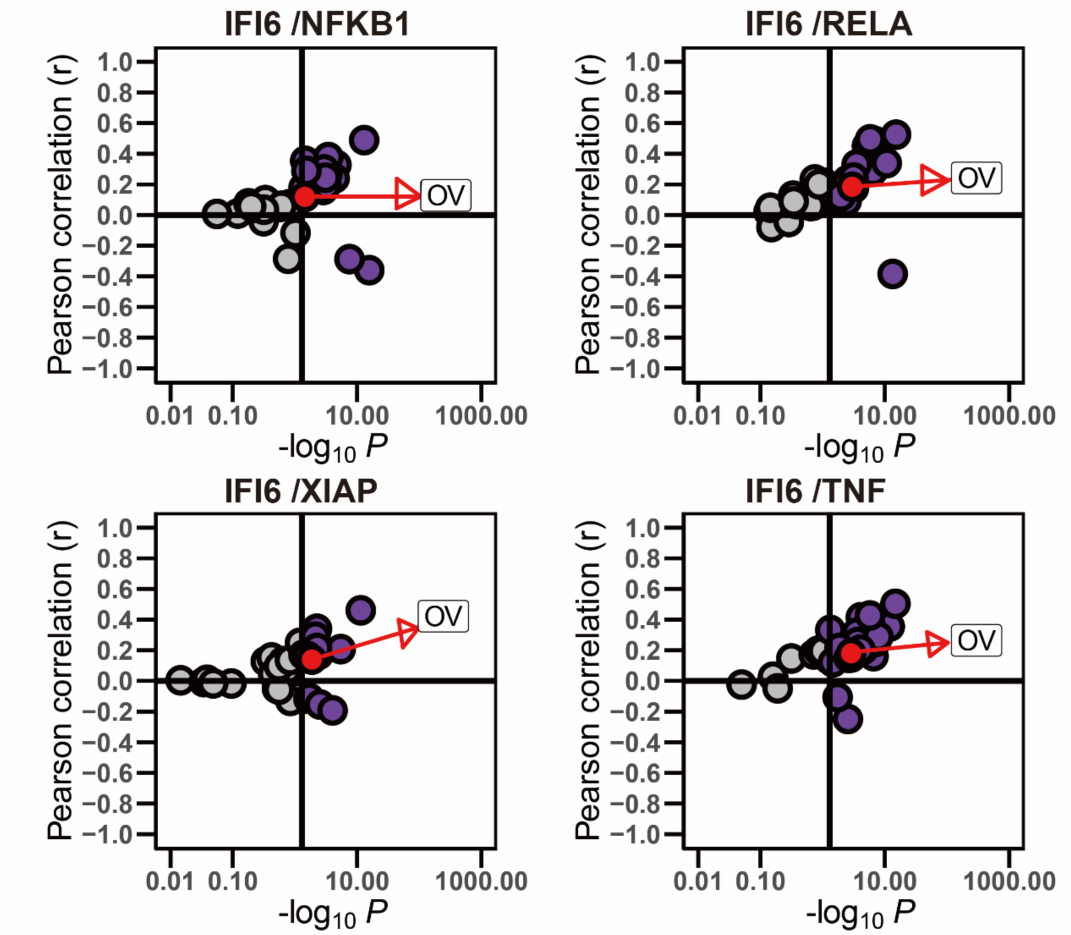

Fig. S8 Calibration plots for Riskscore based on TCGA OC dataset and GEO OC meta_dataset.

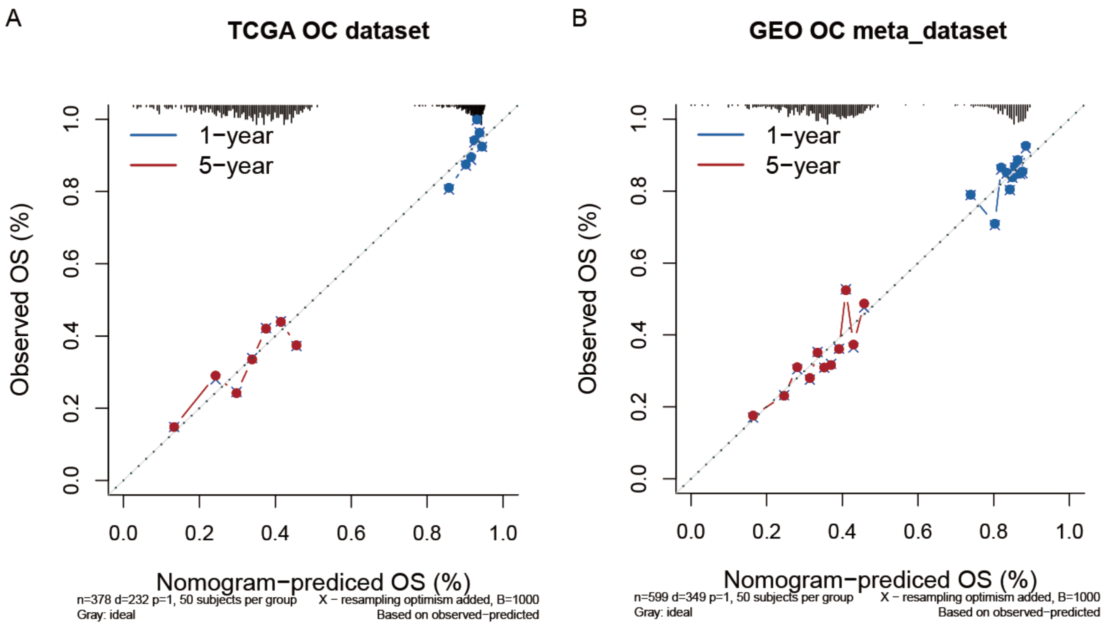

Table S1. All the datasets used in the manuscript.

A. The Cancer Genome Atlas (TCGA) datasets download from UCSC Xena.

Cancer	Samples
Acute Myeloid Leukemia (LAML)	151
Adrenocortical Cancer (ACC)	79
Bile Duct Cancer (CHOL)	45
Bladder Cancer (BLCA)	430
Breast Cancer (BRCA)	1217
Cervical Cancer (CESC)	309
Colon Cancer (COAD)	512
Endometrioid Cancer (UCEC)	583
Esophageal Cancer (ESCA)	173
Glioblastoma (GBM)	173
Head and Neck Cancer (HNSC)	546
Kidney Chromophobe (KICH)	89
Kidney Clear Cell Carcinoma (KIRC)	607
Kidney Papillary Cell Carcinoma (KIRP)	321
Large B-cell Lymphoma (DLBC)	48
Liver Cancer (LIHC)	424
Lower Grade Glioma (LGG)	529
Lung Adenocarcinoma (LUAD)	585
Lung Squamous Cell Carcinoma (LUSC)	550
Melanoma (SKCM)	472
Mesothelioma (MESO)	86
Ocular melanomas (UVM)	80
Ovarian Cancer (OV)	379
Pancreatic Cancer (PAAD)	182
Pheochromocytoma & Paraganglioma (PCPG)	186
Prostate Cancer (PRAD)	551
Rectal Cancer (READ)	177
Sarcoma (SARC)	265
Stomach Cancer (STAD)	407
Testicular Cancer (TGCT)	156
Thymoma (THYM)	121
Thyroid Cancer (THCA)	568
Uterine Carcinosarcoma (UCS)	56
Pan-Cancer (PANCAN)	10535
GTEX	9783

B. The Gene Expression Omnibus (GEO) OC datasets

GSE ID	Platform	Samples
GSE18520	Affymetrix HG-U133 Plus 2.0 (GPL570)	63
GSE26193	Affymetrix HG-U133 Plus 2.0 (GPL570)	107

GSE30161	Affymetrix HG-U133 Plus 2.0 (GPL570)	58
GSE63885	Affymetrix HG-U133 Plus 2.0 (GPL570)	75
GSE9891	Affymetrix HG-U133 Plus 2.0 (GPL570)	278
GSE54388	Affymetrix HG-U133 Plus 2.0 (GPL570)	22
GSE130000	Illumina NextSeq 500	8
GSE45553	Affymetrix Human Gene 1.0 ST Array (GPL6244)	8
GSE47856	Affymetrix Human Gene 1.0 ST Array (GPL6244)	171

Table S2. 18 genes with statistical significance using the univariable Cox proportional hazards regression based on TCGA OC dataset.

ID	HR	95% CI_low	95% CI_up	P_Value
ATRAID	0.751	0.599	0.941	0.013
CCDC34	0.724	0.589	0.889	0.00201
CHCHD2	0.8	0.651	0.985	0.03532
CLTA	0.731	0.571	0.937	0.01331
COX7A2	0.835	0.71	0.983	0.03026
COX8A	0.833	0.698	0.993	0.0419
DEK	0.801	0.663	0.968	0.02179
HMGN5	0.788	0.664	0.936	0.00663
IFI27	0.924	0.865	0.988	0.0207
IFI6	0.923	0.858	0.992	0.02848
MIF	0.89	0.794	0.998	0.04568
MYL12B	0.782	0.633	0.965	0.02198
NDUFB4	0.805	0.665	0.975	0.02684
NDUFC2	0.786	0.666	0.928	0.00458
OST4	0.804	0.666	0.971	0.02361
SPEN	1.303	1.046	1.622	0.01813
TMEM14C	0.808	0.657	0.994	0.04369
TMEM258	0.741	0.605	0.906	0.00358

Table S3. Gene primer sequence

Gene symbol	Primer sequence (5'--3')
IFI6-F	GGTCTGCGATCCTGAATGGG
IFI6-R	TCACTATCGAGATACTTGTGGGT
NFKB1-F	AACAGAGAGGATTTCGTTTCCG
NFKB1-R	TTTGACCTGAGGGTAAGACTTCT
XIAP-F	ACCGTGCGGTGCTTTAGTT
XIAP-R	TGCGTGGCACTATTTTCAAGATA
RELA-F	ATGTGGAGATCATTGAGCAGC
RELA-R	CCTGGTCCTGTGTAGCCATT
TNF-F	CCTCTCTCTAATCAGCCCTCTG

TNF-R	GAGGACCTGGGAGTAGATGAG

Table S4. Cell number of assigned celltypes across each sample is summarized.

	C0	C1	C2	C3	C4	C5	Total
P1	1591	956	458	193	37	7	3242
P2	986	323	177	20	33	32	1571
P3	1952	77	108	15	19	15	2186
P4	1431	202	250	5	135	62	2085
M1	440	362	112	221	256	98	1489
M2	993	315	33	511	123	51	2026
R1	3953	479	591	80	36	52	5191
R2	2728	279	343	18	46	8	3422
